# Impact of IgA constant domain on HIV-1 neutralizing function of monoclonal antibody F425-A1g8

**DOI:** 10.1186/1742-4690-9-S2-P93

**Published:** 2012-09-13

**Authors:** X Yu, M Duval, C Lewis, L Cavacini

**Affiliations:** 1 Beth Israel Deaconess Medical Center and Harvard Medical School, Boston, MA, USA; 2 Beth Israel Deaconess Medical Center, Boston, MA, USA

## Background

With the majority of HIV infections resulting from mucosal transmission of HIV, induction of an effective mucosal immune response would be pivotal in preventing transmission. HIV-specific IgA but not IgG has been detected in genital tract, seminal fluid, urethral swabs, urine and vaginal wash samples of HIV-negative sex workers and HIV-status discordant couples. Although present at low levels, purified mucosal and plasma IgA from HEPS individuals demonstrated cross-clade neutralizing activity and were able to inhibit HIV mucosal transcytosis.

## Methods

The monoclonal antibody F425-A1g8 was generated in our laboratory by hybridoma technique and showed binding activity to the CD4i site of gp120. We isolated the variable genes of heavy chain (VH) and light chain (VL) from the hybridoma cell line and cloned the VH fragment into the vectors pHC-HuCγ1 and pHC-huCα1 individually, as well as cloned VL into the vector pLC-huCk. Both of VH and VL plasmids were co-transfected into CHO-K1 cells in equimolar amounts and established F425-A1g8 IgG1 and IgA1 expressing cell lines. We characterized the impact of different isotype variants of F425-A1g8 to HIV neutralizing activity by direct HIV viral neutralizing assay and antibody dependent cell-mediated viral inhibition.

## Results

The switching constant domain of F425-A1g8 to construct IgG1 and IgA1 isotypes do not impact their binding activity with the CD4 site binding site of HIV. The result of neutralization showed that in contrast to little neutralization by F425-A1g8 IgG1 in the absence of sCD4, the IgA1 variant of the antibody displayed significant neutralization activity against a range of HIV clade B isolates.

## Conclusion

This research clearly suggests that IgA isotype utilizing its unique molecular structure plays an important role in HIV neutralization. The studies of the neutralizing function of IgA isotypes may also serve to inform the design of vaccine strategies that may be more effective at preventing mucosal transmission.

